# Optimization of SERS Detection for Sulfathiazole Residues in Chicken Blood Using GA-SVR

**DOI:** 10.3390/foods15010134

**Published:** 2026-01-02

**Authors:** Gaoliang Zhang, Zihan Ma, Chao Yan, Tianyan You, Jinhui Zhao

**Affiliations:** 1 Laboratory of Modern Agricultural Equipment and Technology, Ministry of Education, School of Agricultural Engineering, Jiangsu University, Zhenjiang 212013, China; gaoliangzhang2025@163.com; 2 Jiangxi Key Laboratory of Modern Agricultural Equipment, Jiangxi Agricultural University, Nanchang 330045, China; 13518632789@163.com (Z.M.); 13204921315@163.com (C.Y.); 3 College of Agricultural Equipment Engineering, Henan University of Science and Technology, Luoyang 471003, China

**Keywords:** SERS detection, sulfathiazole residues, chicken blood, genetic algorithm-support vector regression modeling

## Abstract

The extensive use of sulfathiazole in poultry farming has raised growing concerns regarding its residues in poultry-derived products, posing risks to human health and food safety. To overcome the limitations of conventional detection methods and address the analytical challenges posed by inherent complexity of chicken blood matrix for the detection of sulfathiazole residues in chicken blood, a rapid and sensitive surface-enhanced Raman spectroscopy (SERS) method was developed for detecting sulfathiazole residues in chicken blood. Four colloidal substrates, i.e., gold colloid A, gold colloid B, gold colloid C, and silver colloids, were synthesized and evaluated for their SERS enhancement capabilities. Key parameters, including electrolyte type (NaCl solution), colloidal substrate type (gold colloid A), volume of gold colloid A (550 μL), volume of NaCl solution (60 μL), and adsorption time (14 min), were systematically optimized to maximize SERS intensities at 1157 cm^−1^. Furthermore, a genetic algorithm-support vector regression (GA-SVR) model integrated with adaptive iteratively reweighted penalized least squares (air-PLS) and multiplicative scatter correction (MSC) preprocessing demonstrated superior predictive performance with a prediction set coefficient of determination (R^2^_p_) value of 0.9278 and a root mean square error of prediction (RMSEP) of 3.1552. The proposed method demonstrated high specificity, minimal matrix interference, and robustness, making it suitable for reliable detection of sulfathiazole residues in chicken blood and compliant with global food safety requirements.

## 1. Introduction

Sulfonamides are among the most extensively utilized classes of veterinary antibiotics in global poultry production due to their broad-spectrum antibacterial activity and cost-effectiveness [1]. In particular, the widespread use of sulfathiazole in poultry breeding has raised concerns regarding its residues in poultry-derived products, posing potential risks to human health and food safety due to the improper use and insufficient withdrawal periods [1,2]. Some regulatory agencies in the worldwide, including the U.S. Food and Drug Administration (FDA) and the European Medicines Agency (EMA), have set stringent maximum residue limits (MRLs) for sulfathiazole in poultry tissues to mitigate antimicrobial resistance and toxicological hazards [2]. Although conventional detection methods such as high-performance liquid chromatography (HPLC) for sulfonamides can offer excellent sensitivity and accuracy, they are associated with several practical limitations. These include laborious sample preparation, reliance on skilled personnel, high costs, and prolonged analysis times [2,3,4]. The analysis time often exceeds 30 min per sample, making them unsuitable for screening or on-site detection scenarios. These limitations highlight the urgent need for developing rapid, cost-effective alternatives for routine monitoring of sulfathiazole residues in chicken blood.

Surface-enhanced Raman spectroscopy (SERS) has emerged as a promising alternative due to its rapid response, high sensitivity and molecular specificity [5]. By leveraging plasmonic enhancement from metallic nanoparticles (e.g., gold or silver colloids), SERS technology can be used to detect trace analytes in complex matrices without the need for extensive purification [6]. Recent studies have demonstrated the feasibility of SERS for detecting sulfonamides in various environmental and food samples, including milk, honey and muscle tissues [7,8]. Its key advantages included minimal sample preparation, rapid analysis (typically 5 to 15 min), and the ability to provide molecular fingerprint information for unambiguous identification [9,10]. However, several challenges impede the reliable detection of sulfathiazole in chicken blood, primarily due to the inherent complexity of the matrix (e.g., proteins, lipids and cellular debris) and the lack of universal colloidal substrates (e.g., gold or silver nanoparticles) tailored for this application.

In this study, we systematically optimized the SERS detection of sulfathiazole in chicken blood by examining two aspects: the colloidal substrate system and detection parameters. Four types of colloidal substrates, i.e., gold colloid A, gold colloid B, gold colloid C and silver colloids, were evaluated for their enhancement performance. Key experimental parameters, including electrolyte type, colloidal dosage, NaCl solution dosage, adsorption time and spectral preprocessing strategies, were systematically investigated to maximize signal-to-noise ratios and minimize matrix interference. Furthermore, a prediction model integrating genetic algorithm (GA) and support vector regression (SVR) was developed to detect sulfathiazole in chicken blood. This approach aims to offer a rapid, cost-effective, and portable method for on-site screening of sulfathiazole residues in chicken blood, aligning with global food safety initiatives.

## 2. Materials and Methods

### 2.1. Reagents and Materials

Chicken whole blood was procured from a local market at Jiangxi Agricultural University in multiple batches to ensure sample diversity and representativeness. Selected chicken blood samples were submitted to a certified testing laboratory for analysis, and the test report confirmed the absence of detectable sulfathiazole residues in the chicken blood.

All chemicals and reagents used were of analytical grade or higher, unless stated otherwise. Sulfathiazole (98%, Shanghai Macklin Biochemical Co., Ltd., Shanghai, China), food-grade sodium citrate (~99%, Shandong Ensign Industrial Co., Ltd., Weifang, China), tetrachloroauric acid trihydrate (≥49.0%, Sigma-Aldrich Trading Co., Ltd., Shanghai, China), silver nitrate (≥99.8%, Sinopharm Chemical Reagent Co., Ltd., Shanghai, China), and formic acid solution (98%, Shandong Keyuan Biochemical Co., Ltd., Heze, China) were used in this study. Anhydrous magnesium sulfate (MgSO_4_, ≥98%), sodium chloride (NaCl, ≥99.5%), calcium chloride (CaCl_2_, ≥99.5%) and acetonitrile solution (≥99.5%) were purchased from Xilong Scientific Co., Ltd., Shantou, China.

### 2.2. Instruments and Equipment

A portable Raman spectroscopy acquisition system was composed of QE65 Pro Raman spectrometer (Ocean Insight, Orlando, FL, USA), 785 nm laser (Ocean Insight, USA), fiber optics, a sampling accessory and a computer. A thermostatic water bath (WB100-4F, JOANLAB Equipment Co., Ltd., Huzhou, China), an electronic balance (FA2004, Shanghai Sunny Hengping Scientific Instrument Co., Ltd., Shanghai, China), an intelligent constant-temperature magnetic stirrer (ZNCL-T, Gongyi Chuanyuan Instrument Manufacturing Co., Ltd., Gongyi, China), a digital ultrasonic cleaner (KQ-500DE, Kunshan Ultrasonic Instrument Co., Ltd., Kunshan, China), a high-speed centrifuge (PK-165, Hunan Pingke Scientific Instrument Co., Ltd., Changsha, China), and a vortex mixer (VORTEX-6, Haimen Kylin-Bell Lab Instruments Co., Ltd., Haimen, China) were employed in the work.

### 2.3. Preparation of Standard Solutions

1 mL formic acid solution was diluted to 1000 mL with acetonitrile solution by thorough mixing to obtain 0.1% formic acid in acetonitrile. 5 mg of sulfathiazole standard was ultrasonically dissolved in 100 mL ultrapure water to yield a 50 mg/L sulfathiazole standard solution. During experiments, the above sulfathiazole standard solution was diluted with ultrapure water to prepare sulfathiazole solutions of various concentrations.

### 2.4. Preparation of Chicken Blood Samples

Firstly, 100 mL of food-grade sodium citrate solution (10 g/L) was mixed with chicken whole blood at a volume ratio of 1:3 (anticoagulant–blood) and stored at low temperature for subsequent use. Next, 8 mL of chicken whole blood was combined with 2 mL of sulfathiazole standard solutions at varying concentrations in a 50 mL centrifuge tube. The mixture underwent vortex mixing for 2 min followed by ultrasonic agitation for 5 min to obtain chicken blood samples containing different concentrations of sulfathiazole. Following this, 2 mL of the prepared chicken blood was combined with 2 mL of 0.1% formic acid in acetonitrile solution. The above mixture was vortexed for 30 s followed by ultrasonic agitation for 10 min and centrifuge at 15,000 rpm for 5 min. The supernatant was collected and filtered through a 0.22 µm membrane for downstream analytical procedures.

### 2.5. Preparation of Colloidal Substrates

The synthesis of gold colloid A was based upon a published protocol with slight modifications [11]. To prepare gold colloid A, a three-necked round-bottom flask containing 150 mL of 2.2 mM trisodium citrate solution was heated to boiling under reflux conditions. Upon reaching boiling, 1 mL of 25 mM HAuCl_4_ solution was rapidly added, resulting in a color change from yellow to pale pink after 10 min. The reaction mixture was then maintained at 90 °C, and 1 mL of 60 mM trisodium citrate solution was immediately introduced. Subsequently, 1 mL of 25 mM HAuCl_4_ solution was added after a 2 min interval. After 30 min, 2 mL of the above solution was withdrawn. After repeating the aforementioned procedure at 90 °C for 13 times, the resulting colloidal solution was cooled to room temperature to yield gold colloid A, hereafter referred to as such throughout this study.

Gold colloid B was synthesized following the modified Frens protocol [12]. 100 mL of 1 mM HAuCl_4_ solution was heated to boiling, and 3.7 mL of 1% trisodium citrate solution was rapidly injected into the boiling solution, followed by continuous stirring and heating for 30 min. Then, the resulting red-brown colloidal solution was cooled to room temperature to obtain gold colloid B.

Gold colloid C was synthesized following a modified protocol [13]. 100 mL of HAuCl_4_ solution (0.01%) was heated to boiling. Subsequently, 1 mL of 1% trisodium citrate solution was rapidly added into the boiling solution, followed by continuous magnetic stirring and heating for 15 min. The resulting colloidal solution was cooled to room temperature to obtain gold colloid C.

A silver colloid was prepared by the method of reference with a slight modification [14]. After 100 mL of AgNO_3_ solution (0.001 M) was magnetically stirred and heated to boiling, 2 mL of 1% trisodium citrate solution was rapidly introduced into the boiling solution. When the above solution changed from colorless to pale yellow, the timing began. After 60 min, the resulting colloidal solution with dark gray was cooled to room temperature to obtain silver colloid.

### 2.6. Raman Spectra Measurement for Samples

A certain volume of colloidal substrates (gold colloid, silver colloid), 20 μL of the prepared sample extract (as described in Section 2.4), and a certain volume of electrolyte solution were sequentially added to the quartz bottle. The mixture was evenly placed in the Raman spectrum sampling attachment, and their Raman spectra were measured after adsorption for a certain time.

The parameters of the portable Raman spectroscopy acquisition system were set as follows: the integration time of 10 s, the laser energy of 800 mW, the average times of 2, the smoothness of 1, the spectral scanning range from 150 to 2100 cm^−1^, and the Raman spectra range of 400 to 1800 cm^−1^ used for data analysis.

### 2.7. Feasibility Test Scheme for SERS Detection of Sulfathiazole in Chicken Blood

To compare the feasibility of SERS detection of sulfathiazole in chicken blood, the SERS spectra of the following samples were measured: gold colloid + NaCl solution, gold colloid + sulfathiazole aqueous solution (10 mg/L) + NaCl solution, gold colloid + blank chicken blood extract (without sulfathiazole) + NaCl solution, gold colloid + chicken blood extract containing sulfathiazole + NaCl solution.

### 2.8. Optimization Test Scheme of SERS Detection Conditions

To examine the effect of different electrolytes on the SERS intensity of sulfathiazole in chicken blood, 550 μL gold colloid, 20 μL chicken blood extract containing sulfathiazole and 60 μL different kinds of electrolyte solutions (0.1 mol/L CaCl_2_ solution, 0.1 mol/L MgSO_4_ solution, 0.1 mol/L KCl solution, 0.1 mol/L NaCl solution and equal volume of ultrapure water) were added to the quartz bottle and mixed evenly. Their SERS spectra were measured after adsorption for 0 min. Five parallel samples were set under each electrolyte solution condition, and their average spectra was taken as the SERS spectra of the sample under this condition. Their SERS intensities at 1157 cm^−1^ were used to determine the optimal electrolyte species for detecting sulfathiazole in chicken blood sample.

To investigate the influence of different colloidal substrates on the SERS intensity of sulfathiazole in chicken blood, 550 μL different kinds of colloidal solutions (gold colloid A, gold colloid B, gold colloid C, and silver colloid) were introduced into quartz vials, followed by the addition of 20 μL chicken blood extract containing sulfathiazole and 60 μL NaCl solution (0.1 mol/L) and mixed evenly. Their SERS spectra were measured after adsorption for 0 min. Five parallel samples were set under each colloidal solution condition, and their average spectra was taken as the SERS spectra of the sample under this condition. By comparing the SERS intensities at 1157 cm^−1^, the optimal colloidal species were determined for the detection of sulfathiazole in chicken blood samples.

To explore the effect of different amounts of gold colloid on the SERS intensity of sulfathiazole in chicken blood, different volumes (450, 500, 550, 600 and 650 μL) of gold colloid, 20 μL of chicken blood extract containing sulfathiazole and 55 μL of NaCl solution (0.1 mol/L) were added to the quartz flask and mixed evenly. Their SERS spectra were collected for 0 min. Five parallel samples were set under the condition of each gold colloid volume, and their average spectra was taken as the SERS spectra of the sample under this condition. The optimal amount of gold colloid for the detection of sulfathiazole in chicken blood was determined by comparing the SERS intensities at 1157 cm^−1^.

To evaluate the effect of different amounts of NaCl solution on the SERS intensity of sulfathiazole in chicken blood, 550 μL gold colloid, 20 μL chicken blood extract containing sulfathiazole, and different volumes (50, 55, 60, 65 and 70 μL) of NaCl solution (0.1 mol/L) were added to the quartz flask and mixed evenly. The SERS spectra were measured for 0 min of adsorption. Five parallel samples were set under the condition of each NaCl solution volume, and their average spectra was taken as the SERS spectra of the sample under this condition. By comparing the SERS intensities at 1157 cm^−1^, the optimal amount of NaCl solution was determined for the detection of sulfathiazole in chicken blood samples.

To determine the effect of different adsorption time on the SERS intensity of sulfathiazole in chicken blood, 550 μL gold colloid, 20 μL chicken blood extract containing sulfathiazole, and 60 μL NaCl solution (0.1 mol/L) were added to the quartz bottle and mixed evenly. The SERS spectra at different adsorption time (0 to 25 min, interval of 1 min) were measured. Five parallel samples were set up under each adsorption time condition, and their average spectra was taken as the SERS spectra of the sample under this condition. By comparing the SERS intensities at 1157 cm^−1^, the optimal adsorption time was determined for sulfathiazole detection in chicken blood samples.

### 2.9. SERS Quantitative Detection Scheme

To establish the SERS quantitative prediction model for the detection of sulfathiazole in chicken blood, 120 chicken blood samples containing sulfathiazole (1 to 38 mg/L) were prepared according to the procedure described in Section 2.4. Among them, 90 samples were randomly selected as the training set samples for establishing the prediction model, and the remaining 30 samples were used as the prediction set samples. Then, under the optimal detection conditions (as determined in Section 3.2), their SERS spectra were measured following the protocol outlined in Section 2.6, and the SERS spectra of 400 to 1800 cm^−1^ were selected to establish the prediction model.

### 2.10. Data Analysis Scheme

The purpose of this paper is to establish a SERS prediction model for detecting sulfathiazole in chicken blood, but its original SERS spectrum is susceptible to interference from many factors. For example, a strong broadband fluorescence background signal is generated from the chicken blood itself and the substrate, submerging the SERS characteristic peaks of the target molecule sulfathiazole. In this paper, we systematically investigated the model prediction effects of various spectral pretreatment methods and their combinations, including adaptive iterative reweighted penalized least squares (air-PLS), air-PLS combined with Savitzky–Golay smoothing (SG), air-PLS combined with first derivative, air-PLS combined with second derivative, air-PLS combined with normalization, air-PLS combined with standard normal variable transformation (SNV), air-PLS combined with multiple scattering correction (MSC). The air-PLS algorithm is implemented in Matlab R2010b, and other spectral preprocessing algorithms are implemented in Unscrambler X 10.4.

In order to screen out the characteristic variables that are most sensitive to sulfathiazole in chicken blood and most resistant to matrix interference from full-spectrum data, and to construct a more robust and more generalized quantitative analysis model, GA was used to select SERS characteristic wavelengths.

The SERS characteristic wavelengths of sulfathiazole in chicken blood were screened by Matlab GA-PLS toolkit. The relevant parameters of GA were set as follows: the initial population of 30, the mutation probability of 0.01, and the crossover probability of 0.5. The fitness function of GA was constructed by RMSECV value, and the iteration was terminated when the iteration is 100 times.

On the basis of the above characteristic wavelength selection of GA, we used SVR to establish a prediction model for sulfathiazole detection in chicken blood using Unscrambler software. The relevant parameters of SVR were set as follows: the type of SVR function of epsilon SVR, the kernel function of radial basis function (RBF), the penalty parameter C of 10, and Gamma of 0.01. In addition, two prediction models, i.e., multivariate linear regression (MLR) and partial least squares regression (PLSR), were established to objectively evaluate the prediction effect of GA-SVR model.

## 3. Results

### 3.1. SERS Analysis of Sulfathiazole in Chicken Blood

**Figure 1:**
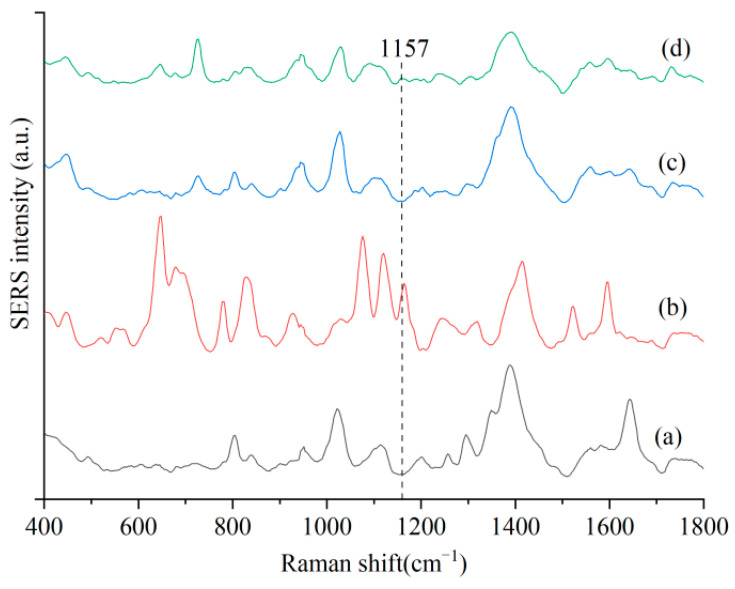
SERS spectrum comparison of chicken blood samples containing sulfathiazole. (**a**) gold colloid + NaCl solution; (**b**) gold colloid + sulfathiazole solution + NaCl solution; (**c**) gold colloid + chicken extraction without sulfathiazole + NaCl solution; (**d**) gold colloid + chicken extraction containing sulfathiazole + NaCl solution.

To validate the capability of the SERS method for detecting sulfathiazole residues in chicken blood, SERS spectra of four sample solutions were compared. As illustrated in Figure 1, the characteristic peak at 1157 cm^−1^ was exclusively observed in the SERS spectra of sulfathiazole aqueous solution and chicken blood extract containing sulfathiazole, whereas no such peak was detected in the SERS spectra of gold colloid + NaCl solution or chicken blood extract without sulfathiazole. Therefore, c can be designated as a specific SERS characteristic peak for identifying sulfathiazole residues in chicken blood.

### 3.2. Optimization of SERS Detection Conditions for Sulfathiazole in Chicken Blood

**Figure 2:**
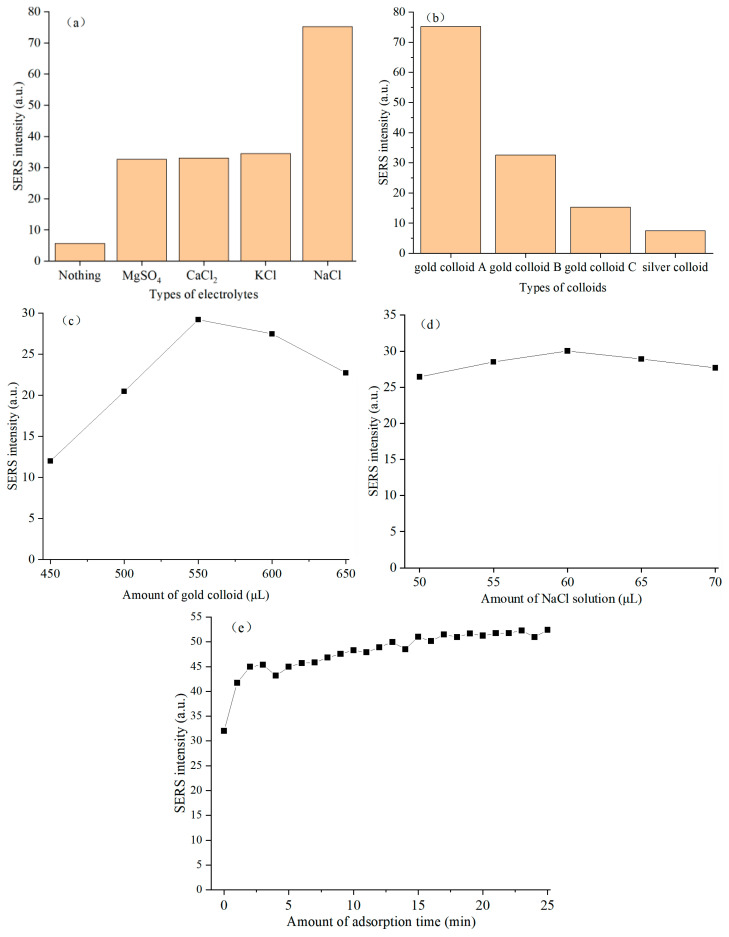
Effects of detection conditions for sulfathiazole in chicken blood on SERS intensity: (**a**) types of electrolytes; (**b**) types of colloids; (**c**) amount of gold colloid; (**d**) amount of NaCl solution; (**e**) adsorption time.

In the SERS detection of complex biological matrices such as chicken blood, the addition of electrolyte is an important factor to optimize the SERS detection sensitivity and overcome the matrix interference. Therefore, the effects of four electrolytes (i.e., MgSO_4_, CaCl_2_, KCl and NaCl) on SERS intensity of sulfathiazole in chicken blood were investigated. Specifically, as shown in Figure 2a, compared with the blank control group without electrolyte, the four electrolytes could significantly enhance SERS intensities of sulfathiazole in chicken blood at 1157 cm^−1^. The SERS intensity of characteristic peak for the blank group was small, indicating that the target molecule sulfathiazole in chicken blood was difficult to effectively approach and adsorb at the SERS active site. The enhancement effect of the four electrolytes was mainly due to their aggregation effect on gold nanoparticles. The ions in the electrolyte could neutralize the charges on the surface of gold nanoparticles, thus inducing the controllable aggregation of gold nanoparticles and generating a strong local electromagnetic field at the gap of gold nanoparticles, which greatly enhanced the SERS intensities of characteristic peak for the sulfathiazole molecule located here [15,16].

As depicted in Figure 2b, the four types of nanoparticle substrates exhibited markedly different SERS enhancement capabilities of characteristic peak at 1157 cm^−1^ for sulfathiazole in chicken blood, which indicated that the type of colloid was a key factor to determine the SERS enhancement effect of sulfathiazole in chicken blood. Specifically, gold colloid A showed the best SERS enhancement performance for sulfathiazole in chicken blood, and its SERS intensity was the highest, which was significantly better than the other three colloids. This indicated that the nanoparticles of colloidal gold A might achieve the optimal configuration in terms of particle size, morphology and aggregation state, and could produce a strong local surface plasmon resonance effect, thus providing the most hot spots and the strongest electromagnetic field enhancement for the target molecule sulfathiazole [17,18]. In summary, gold colloid A was proved to be the best SERS substrate in this system.

It could be seen from Figure 2c that the addition amount of gold colloid had a significant nonlinear effect on the SERS intensity of characteristic peak at 1157 cm^−1^ for sulfathiazole in chicken blood, which indicated that the addition amount of gold colloid was another key factor determining the SERS enhancement effect of sulfathiazole in chicken blood. Specifically, when the amount of gold colloid increased from 450 μL to 550 μL, SERS intensity of characteristic peak for sulfathiazole in chicken blood increased, and reached the maximum at 550 μL. However, when the amount of gold colloid exceeded 550 μL and increased to 650 μL, SERS intensity of characteristic peak of sulfathiazole in chicken blood showed a significant downward trend. In summary, there existed an optimal gold colloid amount of 550 μL in this system, where the formation efficiency of hot spots and the inner filter/aggregation effects reached the best balance, thereby achieving the strongest SERS intensity.

As shown in Figure 2d, different amounts of NaCl solution exhibited a significant nonlinear effect on the SERS intensity of characteristic peak at 1157 cm^−1^ for sulfathiazole in chicken blood, and there existed an optimal amount of NaCl solution. Specifically, when the NaCl solution amount increased from 50 μL to 60 μL, the SERS intensity of the characteristic peak for sulfathiazole in chicken blood increased accordingly, peaking at 60 μL. However, when the NaCl solution amount exceeded 60 μL and increased to 70 μL, the SERS signal intensity showed a downward trend. There existed an optimal amount of NaCl solution of 60 μL in this system, where the controlled aggregation effect and the uncontrolled excessive aggregation effect reached the best balance, thereby achieving the strongest SERS intensity.

As illustrated in Figure 2e, adsorption time significantly impacted SERS intensity of characteristic peak at 1157 cm^−1^ for sulfathiazole in chicken blood, exhibiting a biphasic behavior. One was the rapid adsorption and signal enhancement stage (0 to 5 min), where the SERS intensity of characteristic peak increased sharply with prolonged adsorption time. The second is the adsorption equilibrium and signal stabilization stage (5 to 25 min). The signal growth rate declined markedly after 5 min and stabilized by 14 min. Based on these findings, 14 min was selected as the optimal adsorption time for sulfathiazole detection in chicken blood. At this juncture, the SERS intensity of characteristic peak reached a stable maximum, ensuring both high sensitivity and operational efficiency by minimizing unnecessary waiting periods.

### 3.3. Spectral Preprocessing Results for Sulfathiazole Detection in Chicken Blood

To establish a high-performance quantitative detection model for sulfathiazole in chicken blood, we developed prediction models based on GA-SVR under different spectral preprocessing methods, and systematically compared their predictive performance. All established models achieved a coefficient of determination for the prediction set (R^2^_p_) above 0.90. As shown in Table 1, different spectral preprocessing methods significantly influenced GA-SVR model performance. The GA-SVR model exhibited good performance (R^2^_p_ value of 0.9300, root mean square error of prediction set (RMSEP) value of 3.3956) when air-PLS spectral pretreatment was used alone, demonstrating air-PLS’s effectiveness in eliminating fluorescence and background interference. The first-order and second-order derivative spectral preprocessing methods introduced on the basis of air-PLS were not effective. Among them, air-PLS combined with first derivative spectral pretreatment reduced the number of selected features to 10. Although coefficient of determination for the calibration set (R^2^_c_) and root mean square error of calibration set (RMSEC) were acceptable, R^2^_p_ decreased and RMSEP increased significantly, indicating that the first derivative treatment might lose some effective information related to concentration while improving resolution, which impaired the accuracy of the model. The effect of air-PLS combined with second derivative spectrum pretreatment method was the worst, among which R^2^_c_ and R^2^_p_ were the lowest, while RMSEC and RMSEP were the highest, indicating that the second derivative amplified the noise and destroyed the effective signal in the SERS spectrum to some extent. The spectral pretreatment method of air-PLS combined with MSC had the most prominent effect, which yielded the highest R^2^_c_, lowest RMSEC and second-best

R^2^_p_ with the lowest RMSEP, proving MSC’s ability to preserve concentration-dependent chemical information while mitigating scattering effects. The spectral pretreatment method of air-PLS combined with SNV was slightly inferior to that of air-PLS combined with MSC, but it was still better than that of air-PLS spectral pretreatment alone (RMSEP value decreased from 3.3956 to 3.3220). Air-PLS combined with normalization also showed excellent prediction performance, and its RMSEP was lower than that of air-PLS + SNV and air-PLS + MSC methods, indicating that the normalized spectral pretreatment method had a good effect in adjusting the spectral intensity scale and improving the robustness of model prediction. Compared with air-PLS alone, R^2^_c_ and RMSEC for air-PLS combined with SG spectral pretreatment were slightly improved, but R^2^_p_ decreased and RMSEP increased, indicating that slight over-fitting might be introduced in the SG spectral pretreatment process, resulting in a decrease in model generalization ability. Thus, air-PLS combined with MSC was selected as the preprocessing protocol for subsequent quantitative analysis.

### 3.4. Results of SERS Spectral Feature Extraction for Sulfathiazole Detection in Chicken Blood

**Figure 3:**
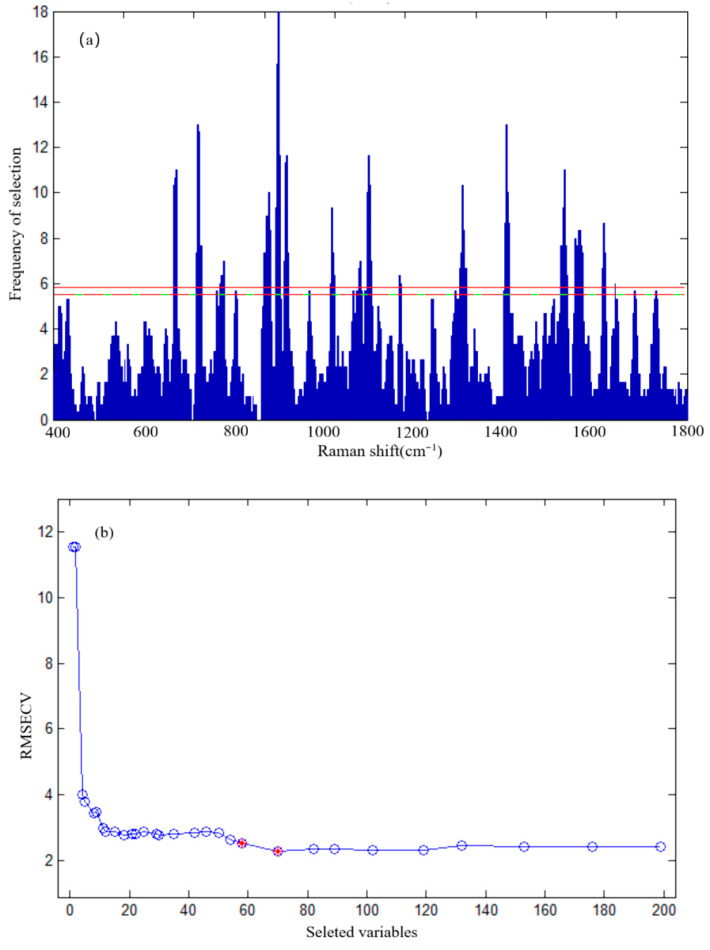
Histogram of frequency of Raman shift selection (**a**) and selected variables (**b**).

To eliminate spectral interference from complex matrices in chicken blood and extract SERS spectral features with the highest specificity and responsiveness for sulfathiazole in chicken blood, we employed GA for SERS spectral feature selection. As shown in Figure 3a, the distribution of selected Raman shifts was obtained across multiple GA ran. The distribution exhibited multiple prominent peaks, indicating that the Raman shifts repeatedly selected by the GA were critical for establishing a predictive model for sulfathiazole in chicken blood. To determine the optimal number of features and evaluate the effectiveness of GA selection, we analyzed the variation trend of RMSECV with the number of selected variables. As shown in Figure 3b, the RMSECV value initially decreased rapidly with increasing variables but gradually stabilized as the number of variables rose. At the initial stage with fewer variables, incorporating more key features significantly reduced RMSECV. However, when the variable count reached approximately 50 to 60, the RMSECV curve entered a distinct plateau phase, suggesting minimal improvement in model performance with additional variables and potential introduction of noise or redundancy that reduced generalization capability. Based on the F-criterion, the algorithm ultimately recommended 58 SERS spectral features for the model.

### 3.5. Results of SERS Predictive Models for Sulfathiazole Detection in Chicken Blood

To establish a rapid quantitative detection method for sulfathiazole in chicken blood, we developed three predictive models, i.e., MLR, PLSR, and SVR. As shown in Table 2, the predictive performance of the three models for sulfathiazole detection in chicken blood exhibited significant differences. Overall, the SVR model demonstrated the highest comprehensive predictive capability, achieving excellent stability and performance across both training and prediction datasets. Thus, SVR was selected for sulfathiazole quantification in chicken blood in this paper.

## 4. Discussion

### 4.1. Optimization Analysis of SERS Detection Conditions for Sulfathiazole in Chicken Blood

Although all of the four electrolyte solutions (i.e., MgSO_4_, CaCl_2_, KCl and NaCl) exhibited enhancement effects, there were significant differences in their enhancement magnitudes, and the order of strength was NaCl solution > KCl solution > CaCl_2_ solution ≈ MgSO_4_ solution. This difference was closely related to the ion type, valence state and characteristics of the electrolyte [15,16]. In addition, in the test sample system of chicken blood, the electrolytes might also slightly denature or precipitate the macromolecules in the tested system through the salting-out effect, reducing their non-specific adsorption on the surface of gold nanoparticles, thus indirectly providing more opportunities for sulfathiazole molecules to contact SERS hot spots. In terms of SERS detection of sulfathiazole in chicken blood, NaCl was confirmed to be the optimal electrolyte type. Its excellent performance was mainly attributed to the synergistic effect of Na^+^ and Cl^−^, which could induce the SERS substrate to form the hot spots of the most suitable high enhancement, and effectively mitigate the interference of chicken blood matrix.

The SERS enhancement effect of gold colloid B and gold colloid C on sulfathiazole in chicken blood decreased sequentially. This trend indicated that even for gold nanocolloids, small differences in their preparation methods or physical and chemical properties, such as particle size distribution, stability and zeta potential, would greatly affect the aggregation behavior of nanoparticles in chicken blood matrix and their interaction with target molecules, eventually leading to a huge difference in enhancement efficiency [19,20,21]. In addition, the SERS enhancement effect of silver colloid on sulfathiazole in chicken blood was the weakest. This might be attributed to the following primary factors. First, silver nanoparticles were prone to uncontrollable agglomeration or surface coating by biological macromolecules in the complex chicken blood matrix, resulting in the loss of plasma characteristics and the decrease in stability [22]. Second, the chemical interaction, such as chemical adsorption, between silver colloid and the target molecule sulfathiazole was weak, and the sulfathiazole molecule could not be effectively fixed in the enhanced region [23].

As shown from Figure 2c, the main reason for the positive enhancement in SERS intensity of characteristic peak at this stage was as follows [24,25]. With the increasing of the number of gold nanoparticles, the number of SERS hot spots formed in the system increased, and the probability of effective interaction with sulfathiazole increased, thereby improving the overall enhancement efficiency. The significant downward trend was mainly due to the following two reasons [26,27]. Firstly, excessive gold nanoparticles were more prone to non-specific and uncontrollable agglomeration in complex chicken blood matrix to form large-sized aggregates, resulting in red shift or broadening of their local surface plasmon resonance characteristics, which reduced the enhancement factor of a single hot spot. Secondly, the high concentration of gold colloid made the laser over-scattered or absorbed when penetrating the measured system, and the generated SERS signal was re-absorbed in the path of the measured system, ultimately resulting in a decrease in the detected SERS intensity of sulfathiazole in chicken blood. In summary, more gold colloids did not necessarily result in better performance in this SERS detection system.

The positive enhancement of SERS intensity of characteristic peak with the increase in amount of NaCl solution was primarily attributed to the following reasons [15,16,28]: an appropriate amount of NaCl neutralized the negative charges on the surface of gold nanoparticles, reduced the electrostatic repulsion between particles, and induced the controlled and moderate aggregation. This aggregation generated a large number of hot spots at the gaps between gold nanoparticles, thereby significantly enhancing SERS intensity of characteristic peak for sulfathiazole. The primary cause for the downward trend with the increase in amount of NaCl solution was as follows [15,16,29]: excess NaCl solution further reduced the zeta potential on the surface of gold nanoparticles, further overcoming the electrostatic repulsion, leading to nonspecific and uncontrolled vigorous aggregation of gold nanoparticles and forming large-sized inactive aggregates. This process would cause redshift or broadening of their localized surface plasmon resonance properties with the enhancement factor of individual hot spots decreasing sharply. Meanwhile, large aggregates might precipitate from the system solution, further reducing surface area of the effective enhancement. Based on the above reasons, the amount of NaCl solution was a critical parameter for regulating the aggregation state of nanoparticles and optimizing the formation of hot spots in this SERS detection system.

The initial rapid increase in SERS intensity with prolonged adsorption time can be attributed to the abundance of highly active hot spots and unoccupied adsorption sites on the gold nanoparticle surfaces. During this stage, sulfathiazole molecules diffused and rapidly occupied these sites, leading to a significant SERS intensity amplification. The phenomenon of the second stage (5 to 25 min) indicated saturation of available adsorption sites on the gold nanoparticles. As equilibrium was approached, newly introduced sulfathiazole molecules faced the prolonged diffusion times to access residual sites or compete with pre-adsorbed molecules, slowing adsorption kinetics and establishing an adsorption–desorption dynamic equilibrium.

It is noteworthy that biological matrices like chicken blood were highly complex. Some factors such as the concentration of plasma proteins and the degree of hemolysis could potentially affect the aggregation behavior of gold nanoparticles and their interaction with sulfathiazole. In this study, the sample preparation step aimed to minimize such interferences by removing the majority of proteins, cellular debris and other interfering components.

Furthermore, while previous studies have primarily focused on sulfonamides detection of matrices like milk or honey [7,8], our work represents the first comprehensive optimization of SERS detection specifically for sulfathiazole in chicken whole blood. Its successful application in sulfathiazole detection in chicken whole blood demonstrates its potential for clinical and veterinary diagnostic applications.

### 4.2. Analysis of SERS Predictive Models for Sulfathiazole Detection in Chicken Blood

By comprehensive comparison of each evaluation index for spectral preprocessing, air-PLS combined with MSC emerged as the optimal preprocessing method. The resulting GA-SVR model achieved the highest fitness, lowest prediction error, and best precision and stability, confirming its ability to reflect true sulfathiazole concentration variations in chicken blood. Five replicate measurements were performed on chicken blood samples containing sulfathiazole at three concentration levels (12, 28 and 36 mg/L), with relative standard deviations ranging from 2.88% to 6.93%. The average recovery rates of spiked samples were 92.3% to 117.6%. By leveraging the characteristic peak at 1157 cm^−1^, this method could achieve the detection of sulfathiazole in chicken blood with a minimum concentration of 1 mg/L. In addition, future validation should include blood samples collected with different anticoagulants (e.g., EDTA) to assess the adopted method’s broad applicability.

In three models established, R^2^_c_ and R^2^_p_ values of SVR were the highest and most closely matched, indicating strong learning ability and optimal generalization performance, thereby effectively avoiding overfitting of SVR model. But the risk of overfitting was assessed through 10-fold cross-validation and a prediction set (30 samples). Additionally, its RMSEC and RMSEP were the lowest among all models, further confirming minimal prediction errors and high accuracy. The MLR model exhibited exceptional training set fitting (R^2^_c_ value of 0.9900), but its prediction performance deteriorated markedly, with R^2^_p_ dropping sharply to 0.8206 and RMSEP rising from 1.9635 to 5.0826. This substantial gap between training set and prediction set performance was a hallmark of overfitting, suggesting potential overfitting in the MLR model. The PLSR model underperformed compared to the other two models, with the lowest R^2^_c_ and R^2^_p_ values and the highest RMSEC and RMSEP. This indicated that while PLSR excelled in handling collinear data, its predictive capacity for sulfathiazole quantification in chicken blood was limited.

## 5. Conclusions

We systematically optimized SERS detection of sulfathiazole in chicken blood, identifying gold colloid A as the optimal substrate due to its superior enhancement capabilities arising from ideal nanoparticle size and aggregation behavior. Critical parameters were rigorously optimized: NaCl (60 μL) as the electrolyte-induced controlled nanoparticle aggregation, balancing hotspot formation and signal suppression. A gold colloid dosage of 550 μL maximized SERS intensity, while exceeding this volume led to overscattering and signal loss. An adsorption time of 14 min ensured equilibrium between adsorption and desorption dynamics. Spectral preprocessing via air-PLS combined with MSC effectively eliminated fluorescence background interference while preserving chemical information, enabling the GA-SVR model to achieve the highest predictive accuracy (R^2^_p_ value of 0.9278, RMSEP value of 3.1552) among all tested models. The model’s robustness was further enhanced by selecting 58 feature wavelengths through GA, minimizing noise and overfitting risks. The optimized protocol established in this study provides a rapid, cost-effective, and reliable solution for sulfathiazole detection in chicken blood, supporting global food safety initiatives. Furthermore, the proposed methodology could be extended to the detection of other veterinary drug residues in chicken blood, laying a foundation for establishing a comprehensive food safety monitoring system for chicken blood.

## Figures and Tables

**Table 1:** Results of GA-SVR models with different spectra pretreatment methods.

Pretreatment Method	Number of Features	R^2^_c_	RMSEC	R^2^_p_	RMSEP
air-PLS	22	0.9814	1.5863	0.9300	3.3956
air-PLS + first derivative	10	0.9757	1.9473	0.9099	3.5678
air-PLS + second derivative	34	0.8905	3.8487	0.8295	4.8128
air-PLS + SNV	41	0.9833	1.5049	0.9233	3.3220
air-PLS + MSC	58	0.9855	1.3948	0.9278	3.1552
air-PLS + normalization	42	0.9788	1.6965	0.9183	3.3343
air-PLS + SG	25	0.9830	1.5107	0.9003	3.8347

**Table 2:** Prediction performance comparison of different models.

Model	$R_{c}^{2}$	RMSEC	$R_{p}^{2}$	RMSEP
MLR	0.9900	1.9635	0.8206	5.0826
PLSR	0.9667	2.1059	0.7398	5.9243
SVR	0.9855	1.3948	0.9278	3.1552

## Data Availability

The original contributions presented in this study are included in the article. Further inquiries can be directed to the corresponding authors.

## Abbreviations

The following abbreviations are used in this manuscript:

Air-PLS	adaptive iterative penalty least square method
SNV	Directory of open access journals
MSC	multiplicative scatter correction
$R_{c}^{2}$	coefficient of determination for the calibration set
$R_{p}^{2}$	coefficient of determination for the prediction set
RMSEC	root mean square error of calibration set
RMSEP	root mean square error of prediction set
SERS	surface-enhanced Raman spectroscopy
GA	genetic algorithm
SVR	support vector regression
FDA	Food and Drug Administration
MRLs	maximum residue limits
HPLC	high-performance liquid chromatography

## References

[1] Shaaban H., Mostafa A. (2023). Simultaneous determination of antibiotics residues in edible fish muscle using eco-friendly SPE-UPLC-MS/MS: Occurrence, human dietary exposure and health risk assessment for consumer safety. Toxicol. Rep..

[2] Mirzaie S., Jamiri F., Javanmard Dakheli M., Mirdamadi S. (2023). A microbial inhibition assay in microplates using Bacillus licheniformis for detection of enrofloxacin and sulfamethazine in chicken spiked kidney, liver and muscle tissues. Vet. Med. Sci..

[3] Pei J.H., Qi X.H., Zou M.Q., Jin Y., Wang D.Y., Luo Y.J. (2024). Advances in surface-enhanced Raman spectroscopy for the detection of veterinary drug residues in foods of animal origin. Chin. J. Appl. Chem..

[4] Das C., Patel V.D., Gupta D., Mahata P. (2024). Isolation of a Cd-based coordination polymer containing mixed ligands: Time- and temperature-dependent synthesis, sulfonamide antibiotics detection, and schottky diode fabrication. Inorg. Chem..

[5] Bi X., Czajkowsky D.M., Shao Z., Ye J. (2024). Digital colloid-enhanced Raman spectroscopy by single-molecule counting. Nature.

[6] Sun F., Hung H.-C., Sinclair A., Zhang P., Bai T., Galvan D.D., Jain P., Li B., Jiang S., Yu Q. (2016). Hierarchical zwitterionic modification of a SERS substrate enables real-time drug monitoring in blood plasma. Nat. Commun..

[7] Li C.Y., Wang H.Y., Li Y.C., Li J., Chen G.L., Fan Y.X. (2023). Application progress of surface enhanced Raman spectroscopy for detection veterinary drug residues in animal-derived food. Spectrosc. Spectr. Anal..

[8] Wang L.L., Sun F.T. (2024). Application of surface-enhanced Raman spectroscopyin food safety detection. Food Mach..

[9] Zhang L., Wu S., Liu J., Ping M., Yang W., Fu F. (2024). Isolation of aptamers with excellent cross-reactivity and specificity to sulfonamides towards a ratiometric fluorescent aptasensor for the detection of nine sulfonamides in seafood. Talanta.

[10] Ma H., Zhao J., Sun M., He J., Liu J., Mi J., Zhao K., Su J., Tu K., Peng J. (2025). Isotropic shrinkage-inspired strategy for plasmonic nanoparticle-loaded hydrogel sers sensor towards robust and sensitive detection of pesticides. Chem. Eng. J..

[11] Bastús N.G., Joan C., Víctor P. (2011). Kinetically Controlled Seeded Growth Synthesis of Citrate-Stabilized Gold Nanoparticles of up to 200 nm: Size Focusing versus Ostwald Ripening. Langmuir.

[12] Xiong Y., Huang J., Wu R., Geng X., Zuo H., Wang X., Xu L., Ai S. (2023). Exploring Surface-Enhanced Raman Spectroscopy (SERS) Characteristic Peaks Screening Methods for the Rapid Determination of Chlorpyrifos Residues in Rice. Appl. Spectrosc..

[13] He X., Xiong X.D., Liang J.B., Li Z.Y., Zhang X. (2005). Preparation of colloidal gold used in immunoassay and control of particle size. Chin. J. Rare Met..

[14] Sun L.L., Wang C.S. (2020). Highly sensitive and rapid surface enhanced Raman spectroscopic (SERS) determination of thiram on the epidermis of fruits and begetables using a silver nanoparticle-modified fibrous swab. Anal. Lett..

[15] You J.K., Wei X.H., Chen Y.Y., Ye S.M., Yu Y.Y., Zhang H.Y. (2021). Rapid determination of acetamiprid in chinese herbal medicineby visualization method based on aptamer with gold nanoparticles as optical probe. Phys. Test. Chem. Anal. Part B Chem. Anal..

[16] Liu X., Pant U., Logan N., He Q., Greer B., Elliott C.T., Cao C. (2024). Non-linear responses via agglomeration and aggregation of gold nanoparticles for surface-enhanced Raman spectroscopy (SERS) coupled with chemometric analysis for chlorpyrifos detection. Food Chem..

[17] Zhang L., Wang X., Chen C., Wang R., Qiao X., Waterhouse G.I., Xu Z. (2023). A surface-enhanced Raman scattering sensor for the detection of benzo[a]pyrene in foods based on a gold nanostars@reduced graphene oxide substrate. Food Chem..

[18] Alkilany A.M., Murphy C.J. (2010). Toxicity and cellular uptake of gold nanoparticles: What we have learned so far?. J. Nanoparticle Res..

[19] Kuppusamy P., Kim S., Kim S.-J., Park M., Song K.-D. (2023). Sedeveria pink ruby extract-mediated synthesis of gold and silver nanoparticles and their bioactivity against livestock pathogens and in different cell lines. Antibiotics.

[20] Fuller M.A., Köper I. (2019). Biomedical applications of polyelectrolyte coated spherical gold nanoparticles. Nano Converg..

[21] Esposito A., Bonifacio A., Sergo V., Fornasaro S. (2021). Label-free surface enhanced Raman scattering (SERS) on centrifugal silver plasmonic paper (CSPP): A novel methodology for unprocessed biofluids sampling and analysis. Biosensors.

[22] Park H.-Y., Chung C., Eiken M.K., Baumgartner K.V., Fahy K.M., Leung K.Q., Bouzos E., Asuri P., Wheeler K.E., Riley K.R. (2023). Silver nanoparticle interactions with glycated and non-glycated human serum albumin mediate toxicity. Front. Toxicol..

[23] Iancu S.D., Stefancu A., Moisoiu V., Leopold L.F., Leopold N. (2019). The role of Ag+, Ca2+, Pb2+ and Al3+ adions in the SERS turn-on effect of anionic analytes. Beilstein J. Nanotechnol..

[24] Amendola V. (2019). Correlation of surface-enhanced Raman scattering (SERS) with the surface density of gold nanoparticles: Evaluation of the critical number of SERS tags for a detectable signal. Beilstein J. Nanotechnol..

[25] Tahghighi M., Janner D., Ignés-Mullol J. (2020). Optimizing gold nanoparticle size and shape for the fabrication of SERS substrates by means of the langmuir-blodgett technique. Nanomaterials.

[26] Li M., Kang J.W., Dasari R.R., Barman I. (2014). Shedding light on the extinction-enhancement duality in gold nanostar-enhanced Raman spectroscopy. Angew. Chem. Int. Ed. Engl..

[27] Butler H.J., Fogarty S.W., Kerns J.G., Martin-Hirsch P.L., Fullwood N.J., Martin F.L. (2015). Gold nanoparticles as a substrate in bio-analytical near-infrared surface-enhanced Raman spectroscopy. Analyst.

[28] Zhou X., Chen S., Pan Y., Wang Y., Xu N., Xue Y., Wei X., Lu Y. (2023). High-performance Au@Ag nanorods substrate for SERS detection of malachite green in aquatic products. Biosensors.

[29] Li W., Zhang Y., Zhang W., Hu P., Zhang M., Meng X., Zhang X., Shang M., Duan X., Wang C. (2024). Portable SERS-based POCT kit for ultrafast and sensitive determining paraquat in human gastric juice and urine. ACS Omega.

